# Supplementary figures and images for: Automating ACMG variant classifications with BIAS-2015 v2.1.1: algorithm analysis and benchmark against the FDA-approved eRepo dataset
Source: Genome Med. 2025 Dec 12;17:148. doi: 10.1186/s13073-025-01581-y (PMC12706976; doi:10.1186/s13073-025-01581-y)

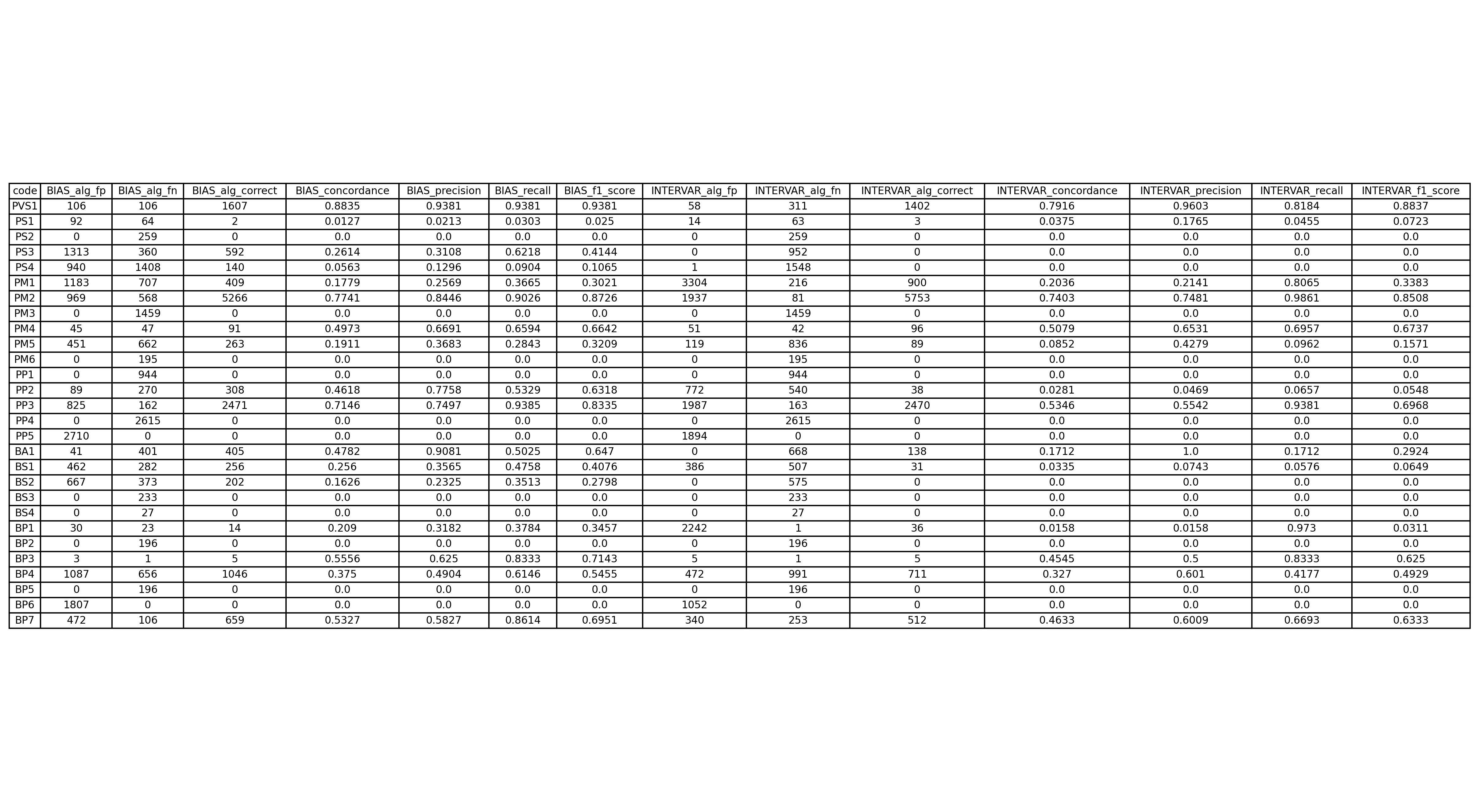

Supplement: Supplementary file 1 — Additional file 1. [file 13073_2025_1581_MOESM1_ESM.png]
